# Disturbed families or families disturbed: a reconsideration

**DOI:** 10.1007/s40519-021-01160-1

**Published:** 2021-03-15

**Authors:** Emma M. Giles, Anastasia S. Cross, Rachel V. Matthews, J. Hubert Lacey

**Affiliations:** 1 Schoen Clinic Newbridge, Birmingham, UK; 2 St Georges, University of London, London, UK

**Keywords:** Carer burden, Parental burden, Anorexia nervosa, Attachment, Emotion regulation, Mentalisation, Family therapy, Parental eating psychopathology

## Abstract

**Background:**

The relationship between anorexia nervosa (AN) and family disturbance has been a subject of debate since its first description. What began as a clear view of the pathologically disturbed family causing AN has become ever more complex over the decades.

**Objective:**

The aim of this review is to explore the literature to examine the changes and evolution of clinical opinion around family dysfunction and AN over the last 20 years.

**Methods:**

A narrative review of heterogeneous studies in peer-reviewed publications sourced from the major databases, including PubMed and ScienceDirect, to illuminate the topic of family distress and AN by highlighting the conflicting and complementary ways it has been studied.

**Results:**

This review has highlighted the complexity of the relationship between anorectic sufferers and their families. It has explored the literature about parental burden, emotions and cognitive mechanisms together with parental attitudes about weight and shape. It is clear that there is no consistent psycho-social pathology in families which has been shown to be causative. However, over the last twenty years, research has highlighted the distress and family dysfunction caused by having to look after an anoretic child with poor mentalisation skills, insecure attachment and emotion dysregulation.

**Conclusion:**

The area has become clearer over the last 20 years; research suggests a bi-directional relationship between AN and family dysfunction, with difficult dynamics becoming entrenched within the family. This is best addressed, the consensus suggests, by specialist family therapy and carer skills interventions. Longitudinal research is needed to definitively answer the question with rigorous scientific certainty.

**EMB rating:**

Level V.

**Level of evidence:**

Level I: Evidence obtained from: at least one properly designed randomized controlled trials; systematic reviews and meta-analyses; experimental studies.

Level II: Evidence obtained from well-designed controlled trials without randomization.

Level III: Evidence obtained from well-designed cohort or case-control analytic studies.

Level IV: Evidence obtained from with multiple time series analysis such as case studies. Dramatic results in uncontrolled trials might also be regarded as this type of evidence.

Level V: Opinions of respected authorities, based on descriptive studies, narrative reviews, clinical experience, or reports of expert committees.

## Introduction

The relationship between anorexia nervosa (AN) and family disturbance has been a subject of debate since Gull and Lasegue first described the modern illness [1, 2]. The association between AN and family dysfunction [3] is generally recognised, but there are differing views on its cause. To some, it is a rational consequence of an increased carer burden of looking after a young person with a disabling physical and mental illness [4]. To others, the association also suggests that poor family functioning is a causal factor for AN. What is accepted amongst clinicians is that a degree of family disturbance is ubiquitous in AN, whilst its role in either the aetiology or maintenance of the disorder is unclear. This review will return to the question first posed by the fourth author [5] nearly twenty years ago. In so doing, it will examine the changes and the evolution of clinical opinion by exploring the literature. The review will try to establish if there is greater professional consensus in professional opinion than there was 20 years ago and greater uniformity on the core question of whether family’s distress is secondary to the illness or is primary and part of the cause of the illness.

Few dispute that AN is a mental health condition which is multifactorial in origin, but has different causal factors operating in each patient [6–8]. The Academy for Eating Disorders position paper emphasises the importance of taking an agnostic view of the relationship between families and aetiology [6]. While there is an association between AN and poor family functioning [3], treatment protocols emphasise that parents should not feel blamed or held responsible for their child’s illness [9]. This is not necessarily led by conviction but by the belief that the lack of blame allows a clinician to look into factors in parental behaviour and family dynamics which may cause or exacerbate the illness. It is important that the association between AN and family functioning is explored as patients frequently report poor family functioning [10, 11] and research has demonstrated that it has a significant impact on treatment outcome [12–14].

This review will explore literature about carer burden, emotion regulation, attachment, and mentalisation. Maternal attitudes about weight and shape will also be explored, as will research into family therapy that pertains to this question.

## Disturbed Families

The parents or carers of children with AN report significantly higher levels of distress than the parents of patients with other mental health conditions, and this applies whether the comparison is a closely related condition such as bulimia nervosa [15, 16] or a quite dissimilar psychiatric disorder such as a psychosis [17]. This high parental burden in itself can lead to parental mental health difficulties, with over 56% of carers of people with AN scoring above the suggestive clinical threshold for anxiety, and 32% for depression [18]. Raenker et al. [19] estimates that 30% of parents present with clinically significant distress. Most parents report feelings of self-blame, isolation, betrayal, and loss which exacerbate carer distress [20, 21]. Carer distress seems to be more pronounced if parents have a personal history of ED, are divorced, have low levels of education, or have pre-existing high levels of anxiety or depression [15, 22].

The reasons for such high levels of carer burden and distress are multiple. Mortality rates in AN are high [23], and often acute physical risks are present [24], which can be alarming and anxiety-provoking for parents [21, 25, 26]. Furthermore, the very nature of AN is intrinsically linked to the family environment: mealtimes become a point of contention, with a “fight for control” over food intake between parents and child [27]. Parents may need to prompt the young person to eat [28], and certain treatments encourage them to take responsibility for mealtimes and weight [29, 30], all of which adds to the parental burden. Arguments over food and weight can lead to parents feeling they are being manipulated and controlled by the illness [20]. Despite this, the anorectic sufferer often does not realise the effect the illness has on parents or the family at large [31].

Parents attempt to understand the causes, consequences and outcomes of AN in a way which is often seen by the child as unhelpful, leading to more maladaptive responses [20, 32, 33]; for example, carers who have a causal misperception and blame themselves for the illness may feel less empowered and demonstrate an over-anxious response. Parental distress and carers’ appraisals can provoke a self-fulfilling cycle, causing more accommodating and enabling behaviours [15, 32], more family conflict and decreased parental alliance [34, 35].

Emotional or cognitive mechanisms shown by young people with AN compound the carer burden for parents, particularly poor mentalisation and problems with emotional regulation. Mentalisation is the ability to understand what is happening in our own and others’ minds [36]. Poor mentalisation and dysfunctional reflective function are reported as common in young people with AN [36, 37]. This can lead to significant interpersonal difficulties and lower emotional connectedness through misunderstandings and ruptures in relationships [38]. Mentalisation difficulties are said to explain why young people with AN report lower perceived social support [39], and significantly lower emotional connectedness compared to their unaffected sisters even before developing the eating disorder [40]. Patients’ descriptions of family dynamics often directly contradict the accounts given by their parents or unaffected siblings [39]. Patients might well be offered support from family members, but have difficulty receiving or understanding it due to their poor mentalisation skills. Unaffected siblings may be protected from family and environmental stressors for they tend to demonstrate less preoccupation with relationships, less need for approval, and more self-transcendence [41].

Poor emotion regulation [42–44] and emotion recognition [42] is frequent in severe and enduring AN, of which poor emotion recognition seems to be a trait and regulation a state [45]. These characteristics can lead to dangerous behaviours, such as self-starving to regulate emotions [44, 46], self-harm [47] and aggression [48]. Understandably, parents can be deeply and negatively affected by these experiences, which also increase their distress [49].

Emotional dysregulation in patients with AN presents a dichotomy: the young person needs support from her or his parents to manage emotions [50], yet struggles to use this due to feelings of anger and impulsivity [51, 52] as well as difficulty in recognising the emotions [42]. This leads to negative emotions being externalised to their body weight and shape potentiating the drift to enduring forms of AN [52]. All this is difficult for parents to understand [33].

Some clinical researchers have put their cards on the table. Holtom-Viesel and Allan [3] suggest the association between AN and poor family functioning should be exclusively viewed as a response to looking after a young person with AN. Sim et al.’s [34] research supports this as they found that when controlling for the emotional impact of AN on the family, there were no differences between families of patients with AN and families of young people with no psychiatric or physical illness. Therefore, it seems that family dysfunction is due to the inevitable complicated family dynamics when parents discover their child has AN [21].

Teaching parents coping strategies to manage the carer distress is beneficial [31]. Skills-based training effectively reduces carer burden [53], increases carer skills and can improve outcomes for the young person [54]. These interventions are effective when delivered by experienced carers [54]. Additionally, supporting parents with anxiety and depression through online CBT reduces their distress and improves reported parent and patient interactions [55].

It is ubiquitous in severe AN, that family life becomes centred around the eating disorder [4]. It is not surprising that the NICE guidelines [56] and other national guidelines recommend family-based work for the treatment of AN. Involving the patients’ family in treatment is suggested for most young people with an eating disorder, but guidelines recommend that the structure should be adapted for each family to best support the recovery [5]. There should also be an emphasis on the multidisciplinary approach [57] and collaborative working with both the patients and carers [58]. Research suggests that understanding parents’ beliefs about AN is likely to be a necessary requirement for successful clinical interventions with families [33].

## Families disturbed

Research into the causes of AN has advanced from the questionable theory of the “psychosomatic family” [59] to acknowledge a multifactorial aetiology, yet it remains imperative to take into account the role families do play in this complex illness. According to Lacoste [60], the two greatest risk factors for AN are family problems and sexual assault. Many would question the latter, few would question the former. Furthermore, poor communication and caring within the family are identified as an adverse interpersonal experience which increases the risk of developing an eating disorder [61, 62].

Issues around attachment to the principal caregivers within the family may be important in the development of an eating disorder [63, 64]. Patients with AN report significantly more emotional detachment from parents than average [63, 65] and greater separation anxiety than their unaffected siblings [66] which, it is argued, leads the young person to develop an insecure attachment style. There also seems to be a high frequency of unresolved trauma and losses in people with AN [67, 68], which is seen as highly relevant to the study of attachment [69]. Research has found that women with an eating disorder are insecurely attached [67, 68, 70, 71], which some argued could be causative in the development of AN: it has been suggested that the eating disorder may be a way for the child to maintain proximity to their parents following the compromise of a secure base [72]. This hypothesis is difficult to test but research has suggested that eating disorder patients with a preoccupied attachment have significant maturity fears [68] which lead them to ‘reject’ mature emotions. This has led to the concept that AN is a retreat from adulthood [73]. Insecure attachments are also strongly related to body dissatisfaction, which is a risk factor for eating disorders [74].

Bruch’s [75] concept that the cause of AN is a deficient sense of self has been revisited [76]. The maturation of self is linked to the development of attachment [77], with attachment insecurity linked to deficits in the self [77, 78]. A neurobiological link has been suggested between the theory of self in the eating disorder and spatiotemporal functioning of the brain [77]. Young people with eating disorders experience distress due to their lack of sense of self, leading to a disconnection from their own emotions and an inability to regulate them [76].

Mentalisation ability has been linked with attachment styles, with research suggesting that secure attachment promotes mentalisation capacity, while insecure attachment and trauma undermine it [38, 79]. The family environment is viewed as critical for the development of mentalisation [80]: maternal mind-mindedness, family discourse, play and maltreatment are all associated with mentalisation development [81]. Rothschild-Yakar et al. [82] name lower mentalisation levels as the most important risk factor in AN and bulimia nervosa.

Parental behaviour can also contribute to emotional dysregulation in a way which becomes instrumental to the development and subsequent maintenance of AN [83]. Emotional dysregulation in parents not only gives their children a poor model of understanding and controlling their emotions, but can also lead them to invalidate their children’s experiences of emotions, thus passing the emotional issues to the next generation [84]. Certainly, emotion dysregulation in young people is associated with early experiences of carers not adequately responding to their feelings [85]. High rates of alexithymia have been found in mothers of children with AN; they are unable to identify and describe emotions experienced by themselves and by others [86]. These high rates of alexithymia are associated with anxiety, neuroticism and depression [86]. This may provide an alternative explanation for the high rates of mental illness in parents of children with AN. These features have been found to be risk factors for eating disorders in their offspring [87]. Some evidence suggests that in anorectic sufferers emotion dysregulation can also be caused more directly, by emotional abuse from parents [83], though such reports are rare and there is no evidence that it is more common in families with an AN child than other populations.

Maternal concern regarding weight and shape does seem, in some, to play a role in the development of disordered eating [62, 88, 89]. A mother with an eating disorder increases the risk of having frank eating disorder psychopathology in a female child, when compared with controls: by age ten, the young person is more likely to diet and hold overvalued ideas about body shape and weight [90]. Other research has indicated that girls younger than 14 years whose mothers had a history of an eating disorder were nearly 3 times more likely than their peers to start purging; however, maternal history of an eating disorder was unrelated to purging in older adolescent females [91]. It is suggested that the family has a more substantial impact in early adolescence, whereas peers are more important in later adolescence and in adulthood [62, 91], due to parents having a major decision-making role in early adolescence [92]. This links with research suggesting that family therapy is less effective when the young person is older in age [93].

Maternal concern regarding weight and shape impacts the family environment [88], which may be causative in the development of AN. The frequency of negative maternal messages has been associated with the young person experiencing weight and shape concerns, exercise fixation, and eating disorder symptoms [89]. Additionally, it was found that when mother–child play interactions were observed, children with mothers with an eating disorder were rated as less involved and less responsive [94]. Parental perfectionism has also been associated with the development of AN [95–97]. Feeding practices can also be impacted; Lydecker and Grilo [98] found that parents with eating disorder psychopathology reported greater perceived feeding responsibility, greater concern about their child’s weight and more monitoring of their child’s eating compared to parents without such characteristics. This study found no significant difference in parents’ restriction of their child’s diet or pressure-to-eat; however, Blisset and Haycraft [99] observed more controlling feeding practices in parents with reported eating disorder symptoms. Maternal feeding practices can vary between siblings and can be part of the non-shared family environment [100]. It seems that addressing weight-related talk, feeding practices and play interactions in the family home may reduce the incidence of disordered eating symptoms in young people. Interventions for these parents are scarce [88] and more research should be done in this area.

Although the case that family dysfunction is causative cannot be made with scientific rigour, it seems probable that it is a maintaining factor in the disease [101], with poor parental mental health and difficult parent–child relationships exacerbating an established carer burden [10, 27]. Treasure et al. [102] found patients’ poor emotional regulation can lead to abusive behaviours towards family members, which is often reacted to with anxiety and avoidance or enabling and accommodating behaviours. It has also been suggested that conflict avoidance within the family may be a maintenance factor [4]. These behaviours perpetuate poor family dynamics and further entrench the eating disorder.

Family relationships [102] and maintenance factors [4] are also important to work on in family-based work. However, prior to beginning family therapy, it is important to assess suitability for treatment, as well as therapeutic alliance, to optimise effectiveness [103]. The personality of the parents should also be considered when engaging the family in psychotherapeutic work, with attention given to the dynamics that specific personality characteristics can create [104]. Research has also suggested that the presence of problematic family behaviours, such as enmeshment and criticism, can lead to lower rates of remission after family therapy [93]. In fact, family involvement can be clinically contraindicated (for example, in the case of severe parental psychopathology), emphasising the need for a thorough assessment [6, 56]. Family therapy should not be restricted to the task of addressing family dysfunction, should it exist; it is a forum in which anorectic sufferers and their families can prepare for change. Most particularly, following recovery, parents lose their emaciated dependent child and receive a newly autonomous person.

Preventative interventions should also be considered to target some difficulties; promising research is being done on interventions that address emotion dysregulation, attachment and mentalisation difficulties in schools [105–108] and in parenting classes [109]. These interventions could help to reduce the risk of family dysfunction and should be researched further.

## Conclusion

This review has highlighted the complexity of the relationship between anorectic sufferers and their families. What began as a clear view of the pathologically disturbed family causing AN has become ever more complex over the decades since the first description 130 years ago. While families are not the primary cause of eating disorders [6], most research in the field is not longitudinal, thus presenting insufficient evidence to definitively answer the question with rigorous scientific certainty.

Over the 20 years, since one of us posed the question [5], certain matters are much clearer. There is no ‘anorectogenic’ mother or family, and no consistent psycho-social pathology in families which has been shown to be causative [4]. Genetic work remains at an early stage but if it did lead to a return of the view that families have a causative role, it would be by a quite different mechanism. Research into genetics may also allow us to be more conclusive about the association between the development of AN and personality characteristics as well as maternal weight and shape concern.

Maternal weight and shape concern, emotional dysregulation, mentalisation difficulties, insecure attachment, family dysfunction and parental mental illness have all been suggested alone or together as causes of AN but the science is unclear. There is more evidence that they maintain an illness already present.

The devastating emotional effect of AN on the family remains ubiquitous. Over the last twenty years research has highlighted the difficulty that parents have in managing an anoretic child with poor mentalisation skills, insecure attachments and emotion dysregulation. There is consensus that carer distress should be a major focus of treatment in younger patients and cannot be ignored at any age. It is important that parents are provided with support, and carer skills interventions are considered, thereby breaking the unhelpful cycle that carer distress creates.

There is greater clinical consensus than there was 20 years ago and greater uniformity on the core question of whether the family’s distress is a secondary result or the primary cause of the illness. The majority view is that there is a bi-directional relationship between AN and the family, with difficult dynamics and vicious cycles becoming entrenched within the family. This is best addressed, the consensus suggests, by specialist family therapy and the various family therapy protocols emphasise that parents should not feel blamed or held responsible for their child’s illness, unless child abuse is known. The lack of blame allows a clinician to look into factors in parental behaviour and family dynamics which may cause or exacerbate the illness and can best lead to recovery. Professionals should be aware of different problematic family dynamics, so that they are equipped to target them on a case-by-case basis to achieve recovery.

This review gives renewed attention to an interesting and important question. The paper has reviewed extensive research to illuminate the conflicting and complementary ways AN and the family has been studied. Important clinical recommendations have also been made. However, based on the original paper, the review is selective in the literature it has included. The paper is not a meta-analysis but it is a meta-review of heterogeneous studies.

This review has explored the literature about carer burden, emotions and cognitive mechanisms together with parental, particularly maternal, attitudes about weight and shape. Evidence from these sources have been used to both implicate and exonerate the parent–child diathesis and the consensus now is to take an agnostic view.

## What is already known on this subject?

In the last 20 years, since one of us posed the question, much research has been done into family dysfunction and anorexia nervosa. It is clear that anorexia nervosa is associated with poor family functioning; however, much of the research is fragmented. This review has analysed the literature to explore this complex association.

## What does this study add?

This review has analysed the literature about parental burden, emotions and cognitive mechanisms together with parental attitudes about weight and shape. It has outlined the complex association between family dysfunction and AN and suggests that there is bi-directional relationship. The review highlights the importance of family therapy and carer/parental skills interventions in the treatment of AN and suggests areas for future research such as preventative measures and interventions for parents with eating disorders.
